# Bacterial Nanocellulose-Enhanced Alginate Double-Network Hydrogels Cross-Linked with Six Metal Cations for Antibacterial Wound Dressing

**DOI:** 10.3390/polym12112683

**Published:** 2020-11-13

**Authors:** Mina Shahriari-Khalaji, Siyi Hong, Gaoquan Hu, Ying Ji, Feng F. Hong

**Affiliations:** 1 Microbiological Engineering and Industrial Biotechnology Group, College of Chemistry, Chemical Engineering and Biotechnology, Donghua University, Shanghai 201620, China; msh072920@gmail.com (M.S.-K.); hgq941115@163.com (G.H.); 2 Scientific Research Base of Bacterial Nanofiber Manufacturing and Composite Technology, China Textile Engineering Society, Shanghai 201620, China; 3 Faculty of Applied Science and Engineering, University of Toronto, Toronto, ON M5S 1A1, Canada; cathyong@163.com; 4 Institute of Textiles and Clothing, Hong Kong Polytechnic University, Hunghom, Kowloon, Hong Kong; ying.ji@polyu.edu.hk

**Keywords:** bacterial nano-cellulose, alginate, cationic cross-linking, pH-responsive, antibacterial activity, wound dressing

## Abstract

Alginate (Alg) and bacterial nanocellulose (BNC) have exhibited great potential in biomedical applications, especially wound dressing. Non-toxicity and a moisture-maintaining nature are common features making them favorable for functional dressing fabrication. BNC is a natural biopolymer that promotes major advances to the current and future biomedical materials, especially in a flat or tubular membrane form with excellent mechanical strength at hydrated state. The main drawback limiting wide applications of both BNC and Alg is the lack of antibacterial activity, furthermore, the inherent poor mechanical property of Alg leads to the requirement of a secondary dressing in clinical treatment. To fabricate composite dressings with antibacterial activity and better mechanical properties, sodium alginate was efficiently incorporated into the BNC matrix using a time-saving vacuum suction method followed by cross-linking through immersion in separate solutions of six cations (manganese, cobalt, copper, zinc, silver, and cerium). The results showed the fabricated composites had not only pH-responsive antibacterial activities but also improved mechanical properties, which are capable of acting as smart dressings. All composites showed non-toxicity toward fibroblast cells. Rat model evaluation showed the skin wounds covered by the dressings healed faster than by BNC.

## 1. Introduction

The advanced wound dressing is a fairly new concept originating in the 20th century [1]. The first and main purpose of advanced dressing is to maintain moisture in the wound bed, as the healing rate is faster with the presence of moist dressing. The second purpose for fabrication of advanced wound dressing is to incorporate other favorable properties to dressing material such as antibacterial activity, high exudate absorption ability, and improved healing rate [2]. Hydrogel dressings are attracting more attention due to their moisture retention capacity and ability to combine with other molecules to produce composite hydrogel dressings to meet different requirements of wounds. In a fast and efficient healing process preventing infection is a critical concern that can begin with germy obstruction when changing the wound dressing [3]. Infection can lead to pain, deep tissue and bone inflammation, chronic wounds, tenderness, cellulitis, abscess, blood poisoning, and even death [4]. Development of novel strategies for the treatment of infected wounds is imperative, and has been highlighted by the World Health Organization (WHO) for several years [5]. Fabrication of an ideal wound dressing that simultaneously provides following features seems to be effective to accommodate a broad range of wound types. The main features include providing a moist environment, absorbing and holding exudate properly for a longer time, easy handling and exchanging, fighting against bacteria, and releasing antibacterial agent into the infected wound bed.

**Scheme 1:**
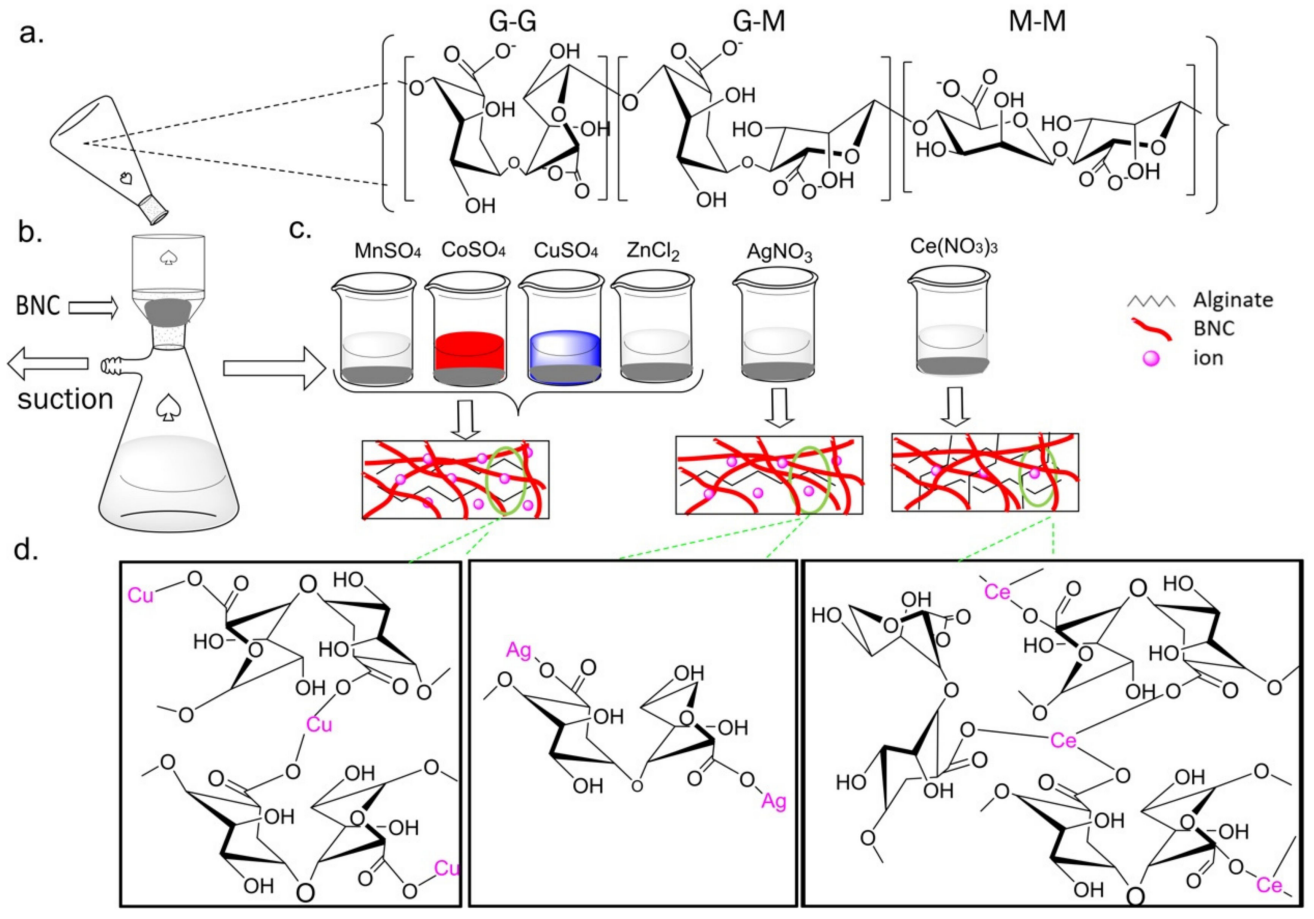
Schematic outline of dressing material fabrication. (**a**): The structure of the repeating units of sodium alginate containing G and M units; (**b**): Insertion of sodium alginate into the bacterial nanocellulose (BNC) matrix using a vacuum suction method; (**c**): Immersing BNC/Alg-Na into six different salt solutions; (**d**): Formation of the composite hydrogels by coordination of different cations between adjacent alginate chains as per the “egg-box” model.

The alginate in seaweeds is extracted by conversion to water-soluble sodium alginate (Alg-Na), which is a natural non-toxic polymer [6,7]. It is a linear and anionic polysaccharide of (1→4) with α-L-guluronate (G) and β-D-mannuronate (M) units under non-regular arrangement (Scheme 1a). Although Alg-Na does not have antibacterial activity, it has an ion-exchange property, which can form antibacterial hydrogel [8,9]. G-rich chains show more tendency toward cationic ions even at low concentration in contrast with M-rich or G and M alternating chains. “Egg box” theory is widely accepted to demonstrate the cation-guluronates interaction (Scheme 1d), which gives the 3D network hydrophilic structure of cation cross-linked alginate hydrogel [10]. It is assumed in present study that the selected cations with three kinds of valence state (monovalent: silver; divalent: manganese, cobalt, copper, and zinc; trivalent: cerium) could affect hydrogel formation differently and bring various cross-linking strength and properties, especially for antibacterial activity, hemostatic activity and wound healing rate.

Even though calcium alginate (Alg-Ca) has exhibited great potential in biomedical applications [11,12], the main drawbacks limiting its potency are lack of antibacterial activity and poor mechanical property, which lead to the fact that alginates generally require a secondary dressing to overcome the defect in weak strength. To address the aforementioned problems and achieve an easy-to-handle composite dressing for clinical treatment, in present study, bacterial nanocellulose (BNC) was used as a support for the primary network, antibacterial alginates were then introduced to form a secondary network via cross-linking with six metal cations in the BNC matrix. Subsequently, a double-network composite hydrogel formed with antibacterial activity. It is hypothesized that the cation-crosslinked composite hydrogel could exhibit favorable mechanical properties, in a metal cation type-dependent manner. Siqueira et al. [13] reported that cellulose nanofibers, derived from plant cellulose, played an important role in improving the dimensional stability of Alg hydrogel. Based on their report, this study aimed to improve water holding capacity (WHC) and water absorption capacity (WAC) with introduction of bacterial nanocellulose as well as to provide a promising pH-responsive antibacterial activity. Pasaribu et al. [14] developed a BNC-based wound dressing impregnated with chitosan and collagen to show antibacterial activities. Yang et al. [15] also developed an antibacterial wound dressing made of BNC and methylglyoxal. Even though the obtained dressing material was tested toward a broad bacterial strain and demonstrated antibacterial activity toward different strains, but no pH-responsive antibacterial activity was reported. Up to now, many antibacterial wound dressings have been developed using different antibacterial agents, but the ability to release more antibacterial agents in the area with lower pH value (an indicator of infected wounds) is a critical concept of functional wound dressing [16,17]. As it is widely reported that pH of infected wound falls to around 5.5 [4], it is expected that the dressing materials could be used as smart dressings due to the possible higher release of cations at lower pH to achieve stronger antibacterial activity. Higher level of cation release can be the result of Alg deformation or shrinkage at lower pH [18]. Calcium cross-linked Alg has been reported to be able to release the calcium ion and turn into a shrunk shape at lower pH than at higher pH [18]. Additionally, the release ratio of copper from sediments was found to be much higher at lower pH than that at higher pH [19], demonstrating a greater tendency towards the release of cations at lower pH.

BNC, which is mainly produced by acetic acid bacteria such as *Komagataeibacter xylinus*, is a natural cellulose material with a unique nanofibrillar and porosity structure [20]. Though having the same molecular formula and no antibacterial activity [21], BNC supersedes vegetal cellulose from the perspectives of great water-holding capability (98–99%), remarkable mechanical properties in wet state, large surface area, high crystallinity and purity, ultrafine network structure, and good biocompatibility [22,23,24,25,26]. BNC-based wound dressings are commercially available for clinical use, exemplified by Dermafill^TM^ (AMD/Ritmed, Tonawanda, NY, USA) [27] and Biofill^®^ and Bioprocess^®^ (Curitiba, PR Brazil) [23].

The development of a functional wound dressings requires multiple consideration to protect the wound site and reconstruct the damaged skin tissues in the wound area. Such design features include antibacterial activity, acceptable mechanical property, great hemostatic activity, no cytotoxicity, moisture maintaining capability, as well as absorbing and holding wound exudates for a long time. This study focused on fabrication of functional composite dressings to fulfill the aforementioned properties, but could also be developed as smart dressings in an infected wound area by stimuli-triggered release of cations. In the present study, six different composite dressings were prepared via introduction of Alg-Na into the BNC matrix through vacuum suction (Scheme 1b) followed by cross-linking with six metal cations (Scheme 1c). The vacuum suction method is a simple, economic, easy, and time-saving technique (only taking 20 min) as compared to the conventional overnight immersion-adsorption method. It is the demonstration to utilize the vacuum suction approach to efficiently incorporate Alg-Na macromolecules into BNC matrix. The properties of dressing materials were evaluated in vitro and in vivo and compared carefully. The obtained dressing materials should demonstrate the design features of ideal wound dressings, while increasing the fluid absorption and holding capacity. In comparison to pristine BNC, improvements in WHC and WAC as well as in antibacterial activity for all the synthesized composites are expected to fulfill infrequent dressing exchange and cost-effective treatment. Furthermore, three commonly applied kinetic models were adopted to elaborate the release profile of the metal cations. To our knowledge, it is the first report to compare the properties of the Algs cross-linked with six metal cations, which presented with three kinds of valence state, and subsequently to synthesize the cation cross-linked Alg/BNC double-network composite hydrogels for evaluation as new functional wound dressing materials.

## 2. Materials and Methods

### 2.1. Materials and Bacterial Strains

*Komagataeibacter xylinus* ATTC 23770 was obtained from American Type Culture Collection, Manassas, VA, USA. *Escherichia coli* (strain 1.1100), and *Staphylococcus aureus* (strain 1.128) were purchased from Institute of Microbiology Chinese Academy of Science (Beijing, China). Fibroblast cells of mouse skin (L929) were purchased from Institute of Biochemistry and Cell Biology of the Chinese Academy of Sciences (Shanghai, China). Sodium alginate (Alg-Na, viscosity (pa.s): >0.02 at 10 g/L and 20 °C), MnSO_4_·4H_2_O, CoSO_4_·7H_2_O, CuSO_4_·5H_2_O, ZnCl_2_, AgNO_3_, and Ce(NO_3_)_3_·6H_2_O were purchased from Sinopharm Chemical Reagent Co. Ltd. (Shanghai, China). Reagents were used without any further purification.

### 2.2. Effect of Cationic Concentration on the Physical Appearance and Hardness of Alginate Hydrogels

To investigate the effect of cationic concentration on the mechano-physical properties of Alg hydrogels, aqueous solutions of MnSO_4_·4H_2_O, CoSO_4_·7H_2_O, CuSO_4_·5H_2_O, ZnCl_2_, AgNO_3_, and Ce (NO_3_)_3_·6H_2_O were prepared respectively at different concentrations (0.5, 0.3, 0.1, and 0.05 M). Firstly 5 mL of Alg-Na solution was transferred into six-well plates, and gently the cationic solutions were added to cover the alginate solution. After shaking at 30 rpm and room temperature for 24 h, the hardness and physical appearance of formed hydrogels were evaluated after washing hydrogel three times with deionized water to remove the excessive cationic ions. For hardness evaluation of hydrogels, the compression measurements were performed according to literature using a universal material testing machine (H5K-S, Houns field Test Equipment Ltd., Redhill, UK) [28]. A single bead was compressed by the probe with a flat end. Measurement of compression was performed at a speed of 0.5 mm/min and 10 N force at 25 °C until up to more than 50% deformation of beads. Each test had eight replications, and mean values ± standard deviations were given.

### 2.3. Preparation of Bacterial Nanocellulose

One colony was picked out from an agar plate of *K. xylinus* and inoculated into a 100 mL liquid seed culture medium and then incubated at 30 °C and 160 rpm. After 18–24 h incubation, each 10 mL of seed culture was transferred into 250 mL flasks containing 100 mL fermentation medium. The culture medium contained glucose 2.5% (*w*/*v*), peptone 0.5% (*w*/*v*), yeast extract 0.3% (*w*/*v*) and pH value was adjusted to 5.0 with citric acid. After static incubation for ten days at 30 °C, BNC membranes were harvested at the surface of each liquid culture medium [20], and washed to remove medium components and microbial cells by repeating the following steps four times: Incubating in 1.0% (*w*/*v*) NaOH aqueous solution at 80 °C for 4 h followed by washing in distilled water for 4 h. Afterward, the BNC membranes were stored in distilled water at 4 °C for further use.

### 2.4. Fabrication of Dressing Material

As shown in Scheme 1b,c, dressing materials were fabricated in two steps. The first step was to dissolve Alg-Na in deionized water to give 1% (*w*/*v*) solution, and then 50 mL of Alg-Na aqueous solution was used to exchange the water in a BNC membrane (7.5 cm diameter, 0.4 cm thickness) that was flat placed on the bottom of a Buchner funnel to obtain a BNC/Alg-Na composite through vacuum suction at 15 kPa. The vacuum was stopped when 10 mL of the Alg-Na solution remained in the funnel to prevent the BNC hydrogel from being over-compressed. The second step was to immerse the BNC/Alg-Na respectively into 50 mL 0.05 M aqueous solutions of MnSO_4_, ZnCl_2_, and Ce(NO_3_)_3_ (colorless solutions), CoSO_4_ (red solution), CuSO_4_ (blue solution), and AgNO_3_ (colorless solution, which turned black in contact with air), with magnetically stirring for 24 h at room temperature. Then the synthesized dressing materials were taken out gently and washed with deionized water three times to remove the excessive free cations. Before starting immersion (the second step), the BNC/Alg-Na material was cut into certain shapes with various sizes using a sharp scissors according to the requirement of each following experiment. For biological tests including antibacterial activity, cytotoxicity test and in vivo healing test, the materials were sterilized by immersing into 75% ethanol for 15 min followed by complete washing with sterile ultra-pure water.

### 2.5. Macro and Micro Morphology of Cation Cross-Linked Alginate/BNC Composites

After immersing BNC/Alg-Na into different cationic solutions, the macro morphologies (size and color) of the synthesized dressing materials were investigated. Micro morphologies of the lyophilized samples, including synthesized cation cross-linked Alginate/BNC dressings, BNC/Alg-Na, and pristine BNC were inspected after gold spraying. The surface characterization was observed using a field emission scanning electron microscope (FE-SEM, FEI Quanta FEG 250) with an acceleration voltage of 15 kV. Image J software (National Institutes of Health, Bethesda, MA, USA) was used to calculate the fiber diameter where 100 single fibers were chosen randomly and used for calculation.

### 2.6. Measurement of Alginate Content in the Alg/BNC Composites

The content of water and alginate in Alg/BNC dressings was obtained using the following formula:

W_B_ = W_E_ − W_D_ (1)

W_C_ = W_A_ − W_D_ (2)

where W_B_ was the water content in the pristine BNC membrane, W_E_ was the weight of water-saturated BNC, and W_D_ was the weight of the same piece after drying at 105 °C for 8 h as dehydrated BNC. Alg solution content (W_C_) was calculated by subtracting W_D_ from the weight of composite dressing after alginate solution insertion (W_A_). A circular piece of Alg/BNC with a radius of 2 cm was used and each test was repeated eight times. The mean values ± standard deviations were given.

### 2.7. Water Holding and Absorption Capacity of Composite Dressings

The water absorption capacity of dressings was measured according to the British Pharmacopeia monograph [23] for Alg/BNC composite dressings. The synthesized dressings were cut into a rectangular shape with a size of 3 cm × 2 cm and were weighted as W_0_. W_1_ was the weight of the same sample after freeze-drying followed by soaking into an aqueous solution containing a mixture of 2.5 mmol/L CaCl_2_ and 142 mmol/L NaCl for 30 min. W_2_ was the weight of the dressing samples when placed on cotton gauze and centrifuged at 1200 rpm for 15 min. The cotton gauze was put on the bottom of a centrifuge tube to absorb water. W_3_ was the final weight of completely dried dressings at 105 °C overnight. Water holding capacity (WHC) and water absorption capacity (WAC) of the dressings, water held within the fiber (WBF), and distribution of water within the material (WDWM) [6,28] were calculated using the following formula. Each test was repeated eight times at 25 °C and 40% relative humidity. Mean values ± standard deviations were given.

WHC = (W_0_ − W_3_)/W_3_ (3)

WAC = (W_1_ − W_3_)/W_3_ (4)

WBF = (W_1_ − W_2_)/W_3_ (5)

WDWM = (W_1_ − W_2_)/(W_2_ − W_3_) (6)

### 2.8. Antibacterial Activity

Antibacterial activities were determined using the absorption method according to ISO20743-2007 standard against *E. coli* and *S. aureus* as the models of Gram-negative and Gram-positive bacteria, respectively. A germy inoculum (1 × 10^5^ to 3 × 10^5^ CFU/mL) of 0.25 mL was directly placed on each piece of sterile pristine BNC control and synthesized dressings. After touching with the samples for 24 h, bacterial cells were washed out of the samples with 20 mL of autoclaved Soya Casein Digest Lecithin Polysorbate (SCDLP) medium using a vortex mixer. The SCDLP broth medium was composed of 17 g/L casein peptone, 3 g/L peptone from soy, 2.5 g/L KH_2_PO_3_, 5 g/L sodium chloride, 2.5 g/L glucose, 7 g/L polysorbate 80, 1 g/L lecithin, and pH was adjusted to 7.2 ± 0.2 with sodium hydroxide. The number of viable bacterial cells from the samples was quantified by using spread plate count method after serial dilutions. Each sample had three parallels, and the logarithmic mean values of bacterial colonies and standard deviations were given.

### 2.9. Mechanical Properties

All BNC/Alg samples were cut into rectangles of 1.5 cm × 5 cm. A universal material testing machine (H5K-S, Houns field Test Equipment Ltd., Redhill, UK) was used to evaluate the tensile stress of dressing materials under ambient condition (25 °C, around 40% relative humidity) at a constant cross-head speed of 50 mm/min with a load capacity of 100 N until rupture. Each test was repeated three times.

### 2.10. pH-Responsive Release of Cations

The release of cations was characterized and recorded for 48 h using an inductively coupled plasma-mass spectrometer (ICP-MS, Agilent 7500ce, Tokyo, Japan) [4]. The synthesized dressings were cut into round discs with a diameter of 3 cm. The discs were immersed into 10 mL of phosphate buffer saline (PBS) at pH of 5.5, 7.4, and 8.4, then incubated under agitation at 40 rpm and 37 °C for 12, 24, and 48 h, respectively. At each time point, the 10 mL of buffer was collected for measuring the released cationic ions, and replaced by another 10 mL of fresh PBS. Cross-linking density was also reflected based on the accumulative release of metal cations after 48 h, at pH 7.4 and reported as mg/cm^2^.

### 2.11. Kinetic Analysis of Cation Release

The release profiles of the cations were fitted using the kinetic models commonly applied in drug release studies [28,29,30,31,32]. The models used, Higuchi model (7), Zero-order kinetic model (8) and First-order kinetic model (9) are as follows:

Q_t_/Q_f_ = K t ^1/2^ (7)

Q_t_/Q_f_ = K t (8)

Ln (1- Q_t_/Q_f_) = −K t (9)

where Q_t_ is the release amount of cation at time t (mg/mL), Q_f_ is the final release amount of cation (mg/mL), t is the release time (min), and K is the kinetic constant. The fitness was evaluated using the correlation coefficient, r^2^.

### 2.12. In Vitro Whole-Blood Clotting Evaluation

The whole-blood clotting of the dressings was tested according to literature [33,34,35]. To investigate the effect of synthesized material on the blood clotting, a piece of sterile synthesized dressings (disc shape with a diameter of 1 cm, n = 3) was placed in a tube and pre-warmed at 37 °C, followed by the addition of 100 μL of recalcified whole-blood (20 µL of 0.25 M CaCl_2_ in 2 mL blood of rat) into each tube to directly contact dressing materials. The tubes were incubated for 5, 15, 25, 35, and 45 min at 37 °C. After the pre-set time period, 1 mL of deionized water was added gently to release unbound blood without disturbing the clot that formed on the surface of dressing materials. The absorbance of the collected supernatant was recorded at 550 nm by using a microplate reader (Molecular Devices). Three replicates were performed. The absorbance of 100 μL of recalcified whole blood on the surface of pristine BNC was measured as a negative control.

### 2.13. In Vitro Hemolytic Rate

Hemolytic rates of the synthesized dressings were measured, referring to literature [32,35,36]. Erythrocytes were obtained by centrifuging the whole blood for 10 min at 115× *g* at room temperature and washed three times with PBS gently. Then the washed erythrocytes were diluted to a final concentration of 5% (*v*/*v*). Before that, sterile dressings were placed in 24 well plates and immersed into the normal saline solution for 2 h and kept at 37 °C. Saline solution was removed and 1 mL of the following solutions were added to wells: Deionized water to the positive control, sodium chloride solution to the negative control, and samples. Then 500 μL of the 5% (*v*/*v*) erythrocyte suspension was added into each well, and then incubate at 37 °C for another 2 h. Finally, 1 mL of the supernatant was gently removed by pipette and transferred into new tubes. All the tubes were centrifuged at 115× *g* for 15 min. The supernatant of each tube was gently transferred into a new 96-well plate. The absorbance of the solutions was read at 550 nm using the microplate reader.

The hemolysis of the synthesized dressing was calculated using the equation:

Hemolytic ratio %= (OD_s_ − OD_n_)/(OD_p_ − OD_n_) × 100 (10)

where OD_s_ was the absorbance value of supernatant from the sample groups, OD_p_ was the absorbance value of the positive control, and OD_n_ was the absorbance value of negative control. Each group performed in triple technically and biologically.

### 2.14. Cytotoxicity Test of Composite Dressings

In order to investigate the effect of the dressings on the viability and proliferation of L929 fibroblast cells, a piece of sterile dressing (disc shape with a diameter of 1 cm, n = 3) was placed in a well of a 24-well plate to cultivate cells on the surface of dressings. L929 fibroblast cells of 1 × 10^4^ cells/well were seeded on the surface of each dressing and deepened in the complete culture medium, Dulbecco’s Modified Eagle Medium (DMEM) with 10% of Fetal Bovine Serum (FBS), and then incubated at 37 °C in a humidified 5% environment. The culture medium was changed every 24 h. Cytotoxicity was measured by using Cell Counting Kit-8 (CCK-8). After removing the culture media and washing the cells three times with PBS, 400 µL of DMEM supplemented 10% of CCK-8 reagent were added to each well of 96 well plates to measure CCK-8 absorbance. After incubation for 1 h, the absorbance of 100 µL supernatant from each well (with three parallels) was measured at 450 nm on days 1, 3, and 5. The amount of the orange-colored formazan dye generated by cellular dehydrogenase is directly proportional to the number of living cells. The 24-well plate was used for proliferation of L929 fibroblast cells, and the 96-well plate was used for CCK-8 analysis.

### 2.15. In Vivo Healing Test

All 350 g male rats were selected randomly, fed with a standard diet, and acclimatized for one week before surgery in the lab (24–27 °C). The in vivo experiment conducted strictly following the ARRIVE guidance [32] and the norms given by the Laboratory Animal Ethics Review Committee of Donghua University (DHUEC-NSFC-2019-13). The relevant experimental protocols were performed according to Guides for the Care and Use of Laboratory Animals (Ministry of Science and Technology of China, 2006). For the surgery section, all procedures were performed under aseptic conditions. Rats were anesthetized via injection of chloral hydrate at the dosage of 0.4 mg/kg body weight. The dorsal region of the rats was shaved and sanitized with 75% ethanol as surgery preparation. Full-thickness wounds in a square shape with 5 mm × 5 mm size were generated on either side skin of the spinal line with each 3 cm interval. The wounds were dressed by 2 cm × 2 cm of sterile dressing materials. The dressing materials were changed daily with the same neat sterile dressings. After surgery, the rats were monitored until the 12th day, and the photos were taken periodically. Wound healing (%) was calculated according to literature [29] using the following equation:

wound healing% = (Area_0_ − Area_n_)/Area_0_ × 100% (11)

where Area_0_ was the wound area on day 0, and Area_n_ was the wound area on day n (in a unit of mm).

### 2.16. Statistical Analysis

All analytic statistics were reported in this work as a mean value ± standard deviation (SD). In the analysis of the statistical analysis, the statistical significance was tested by one-way analysis of variance (ANOVA). Statistical difference was considered significant with a *p*-value of less than 0.05.

## 3. Results and Discussion

### 3.1. Effect of Cation Concentration on Appearance and Hardness of Alginate Hydrogels

**Figure 1:**
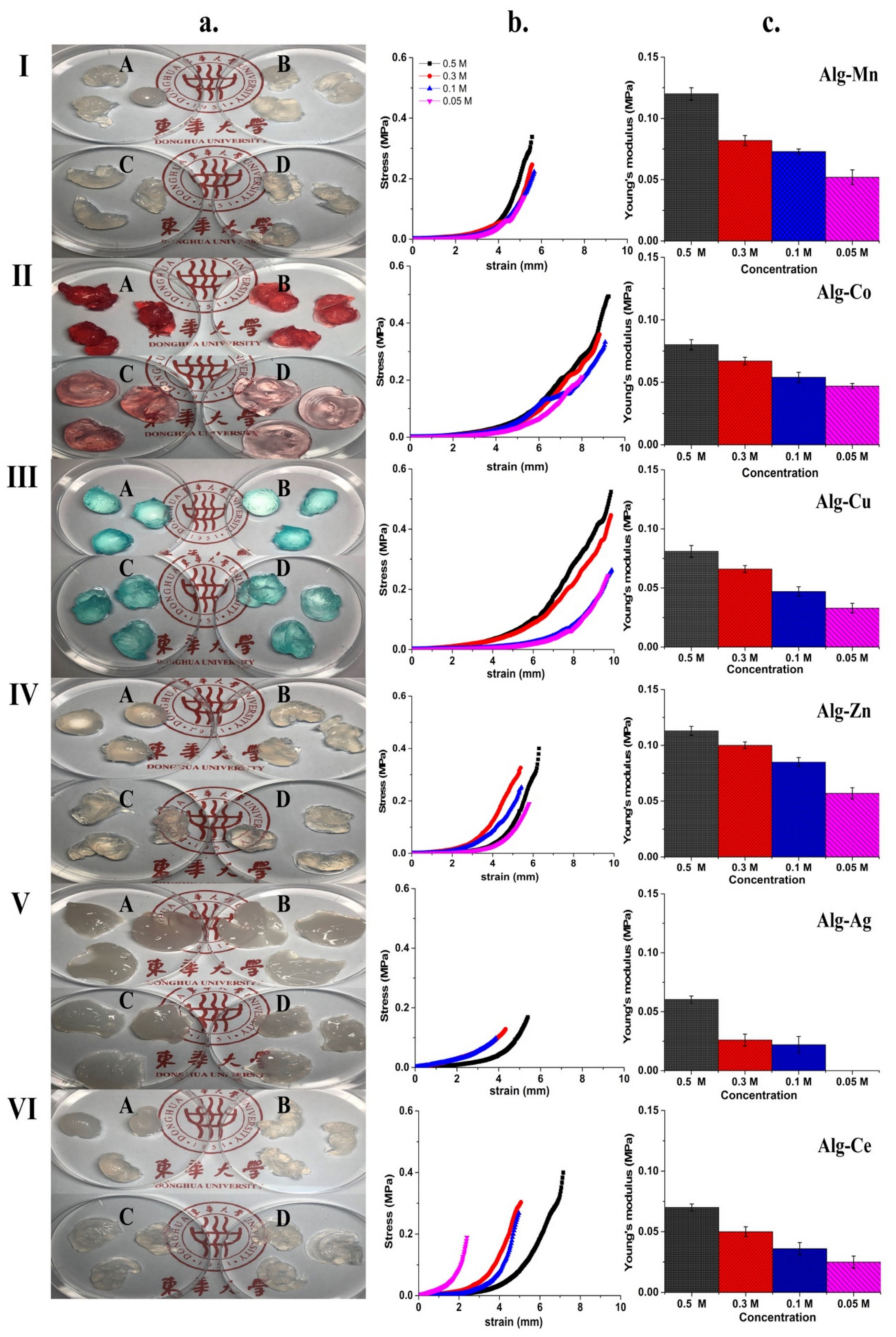
(**a**) Morphology of hydrogels of (I) Alg-Mn, (II) Alg-Co, (III) Alg-Cu, (IV) Alg-Zn, (V) Alg-Ag, and (VI) Alg-Ce at cationic concentrations of (A) 0.5, (B) 0.3, (C) 0.1 and (D) 0.05 M, as well as (**b**) corresponding compressive strength and Young’s modulus of hydrogels (**c**).

By adding each cation into a sodium alginate aqueous solution of 5 mL, cation cross-linked Alg hydrogels formed immediately, and the hydrogels showed the concentration-dependent property (Figure 1). The hardness of all hydrogels made with the same cation, increased with the increase of cation concentration. All cation cross-linked Alg materials showed uniform and three-dimensional hydrogel formation (Figure 1a). The hardness measurement demonstrated that the compressive strength for the deformation of Alg-Cu hydrogel was greater than other cation cross-linked Algs, descending in the following order: Alg-Cu > Alg-Co > Alg-Ce > Alg-Zn > Alg-Mn > Alg-Ag at 4 cation concentrations (Figure 1b). Strain indicated the shape deformation up to 50%. The Alg-Cu showed the highest strain value followed by Alg-Co > Alg-Zn > Alg-Mn > Alg-Ce at the cation concentration of 0.05 M. The alginate hydrogel containing silver ion at 0.05 M concentration of AgNO_3_ was too soft to bear any compression presser (V, Figure 1). The Young’s modulus of hydrogels was also calculated using stress-strain carve presented in Figure 1c.

The properties of alginate hydrogels have been found to be highly influenced by cationic concentration, indicating that a more rigid hydrogel formed with a higher concentration of cationic ion, as described in literature [37,38]. It has been reported that sodium alginate aqueous solution can form hydrogel through the addition of some cationic ions including Mn^2+^, Co^2+^, Cu^2+^, Zn^2+^, Ag^+^, and Ce^3+^ because of its ion-exchangeability [8,37,39,40,41,42]. According to the requirement on highly desired properties of a functional wound dressing material, a suitable cross-linking cation concentration for hydrogel formation must be chosen in order to provide a proliferation of fibroblast cells and promote antibacterial effect. After comprehensively considering the possibility for hydrogel formation, the suitable hardness for dressing, as well as good antibacterial activity, 0.05 M was finally selected as the cationic concentration for further study on BNC-based composite dressing.

### 3.2. Macro and Micro Morphology of Different Cation Cross-Linked Alg/BNC Composites

**Figure 2:**
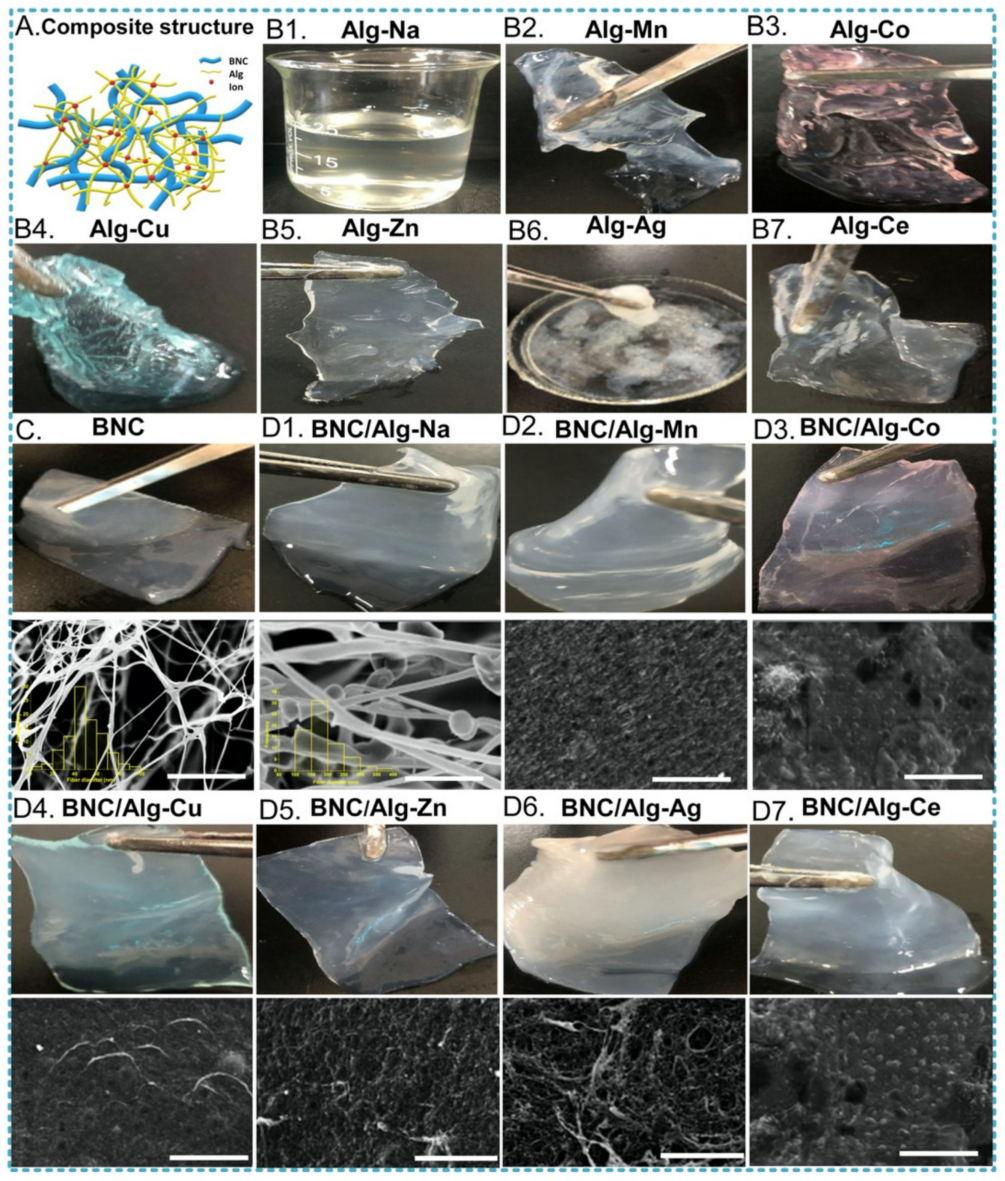
Schematic diagram of metal cation cross-linked Alg/BNC composite hydrogels with the double-network structure (**A**), visual image of Alg-Na aqueous solution (**B1**) and cation cross-linked Alg hydrogels (**B2**–**B7**), visual and FE-SEM images of pristine BNC membrane (**C**), as well as visual and FE-SEM images of cross-sections of BNC/Alg-Na (**D1**) and metal cation cross-linked Alg/BNC composite hydrogels (**D2**–**D7**). The scale bar is 2 μm (amplification: ×20k).

Sodium alginate can exchange sodium with other metal cations to form a water-insoluble hydrogel. The cation cross-linked Alg hydrogels showed a fragile and non-uniform structure (Figure 2(B2–B7)), which clearly indicated the poor mechanical property that is difficult to measure. After immersing BNC/Alg-Na into different cation solutions, the macro morphology (size and color) of the BNC/Alg composites changed. The size of the BNC/Alg-Na piece after immersed into Cu^2+^ solution became much smaller than that before immersion and pristine BNC. The size of dressing materials reached 21.0 ± 0.3, 19.5 ± 0.3, 19.0 ± 0.2, 18.4 ± 0.3, 17.4 ± 0.4, and 13.4 ± 0.3 mm, respectively for BNC/Alg-Ag > BNC/Alg-Mn > BNC/Alg-Ce > BNC/Alg-Zn > BNC/Alg-Co > BNC/Alg-Cu, in comparison to BNC/Alg-Na (22.1 ± 0.5 mm). The size change of composites shows the degree of cross-linking. It has been reported that the tendency toward different metal cations is distinct for alginate, and the obtained result is in compliance with the suggested order in literature [28]. The pristine BNC obtained from *K. xylinus* ATCC 23770 strain showed semitransparent appearance (Figure 2C) and kept the state after Alg-Na incorporation (Figure 2(D1)), but after cross-linking different cations, BNC/Alg composites showed the color which came from metal cations (Figure 2(D2–D7)), for instance, BNC/Alg-Co showed red color due to the presence of Co^2+^. The color of BNC/Alg-Cu was blue because of Cu^2+^. BNC/Alg-Ag was white, which turned light black in contact with air. BNC/Alg-Zn, BNC/Alg-Mn, and BNC/Alg-Ce showed light white color. All obtained cation cross-linked Alg/BNC showed a nonfragile nature, which is easy to handle during application.

The morphology observation by FE-SEM showed porous structure in pristine BNC with an average fiber size of 46.69 nm (Figure 2C). Alg substance/mass was found in the pores of BNC network and coated on BNC nanofibers after Alg-Na insertion by vacuum suction followed by freeze-drying. The average fiber size was subsequently increased to 189.91 nm after Alg insertion (BNC/Alg-Na, Figure 2(D1)). The fiber diameters became thicker in BNC/Alg, and the homogeneous distribution of Alg-Na was observed in the cross-section of BNC membrane. The FE-SEM images of all the cation cross-linked Alg/BNC composites exhibited a uniform distribution of Alg hydrogel into the pores of BNC, and no fibril network could be visible as that in the pristine BNC and that before cation cross-linking (Figure 2(D2–D7)). This is because the amplification of 20,000 was not enough though a second network formed after cation cross-linking alginate macromolecules in addition to the first network of pristine BNC. Schematic diagram of metal cation cross-linked Alg/BNC composite hydrogels with the double-network structure is shown in Figure 2A.

### 3.3. Water and Alginate Content in Dressing Material

The weight of water-saturated BNC was 3.35 ± 0.15 g, which contained 3.33 ± 0.08 g water and 0.024 ± 0.001 g dry BNC. The wet weight of the same piece of BNC after Alg-Na insertion was 2.85 ± 0.01, containing 0.024 ± 0.001 g dry BNC and 2.83 ± 0.13 g 1 *w*/*v*% Alg-Na solution. The result showed that under vacuum suction (at 15 kPa), 85% of the initial weight of the water in the water-saturated BNC was replaced by 1 *w*/*v*% Alg-Na solution, which filled the pores in the BNC network. As the viscosity of Alg-Na is higher than water, the BNC membrane lost 15% of water without Alg replacement. The FE-SEM images confirmed the result after Alg insertion by vacuum suction.

### 3.4. Water Holding and Absorption Capacity of Dressings

As shown in Table 1, the WHC of the synthesized dressing materials reached 42.25, 23.34, 20.16, 23.66, 49.55, and 37.38 g/g for BNC/Alg-Mn, BNC/Alg-Co, BNC/Alg-Cu, BNC/Alg-Zn, BNC/Alg-Ag, and BNC/Alg-Ce, respectively, in comparison to 20.54 g/g of pristine BNC, indicating an improvement of the WHC in most of the synthesized dressing materials (*p* < 0.01 in the case of BNC/Alg-Mn, BNC/Alg-Co, BNC/Alg-Zn, BNC/Alg-Ag, BNC/Alg-Ce, and *p* > 0.05 for BNC/Alg-Cu).

WAC, indicated by the gram water per gram mass of the fluid absorbed by dressing material, is a vital factor in absorbing wound exudates and fluid in wound bed to maintain a moist and neat environment in wound area. The WAC of the freeze-dried hydrogel achieved 31.38, 19.26, 17.51, 21.26, 46.52, and 33.89 g/g for BNC/Alg-Mn, BNC/Alg-Co, BNC/Alg-Cu, BNC/Alg-Zn, BNC/Alg-Ag, BNC/Alg-Ce, respectively, in comparison to 15.24 g/g of pristine BNC, indicating an improvement of the WAC in all the composite dressing materials. The WAC of the dressing materials cross-linked with Mn^2+^, Ag^+^, and Ce^3+^ (*p* < 0.01) increased up to two-fold, whereas the WAC of the materials cross-linked with Cu^2+^ (17.51 g/g) was closest to that of BNC (15.24 g/g) (*p* < 0.05).

WBF determines the ability to maintain proper moisture incitement in wound area [18]. Except for BNC/Alg-Co and BNC/Alg-Cu, the WBF of BNC/Alg-Mn, BNC/Alg-Zn, BNC/Alg-Ag, and BNC/Alg-Ce showed better results than Tegagel (WBF: 18.6 ± 0.81, 3M company), which is a commercially available dressing of alginate [6], and the WBF of all dressing materials was higher than that of pristine BNC.

WDWM reflects the fluid distribution within wound dressing materials. The WDWM of BNC/Alg-Ce, BNC/Alg-Zn was higher than that of Curasorb (8.62 g/g of WDWM), which is another commercially available alginate dressing, and the WDWM of all the synthetic dressings was better than that of Tegagel (WDWM:1.75, 3M company) [6].

### 3.5. Antibacterial Activity

**Figure 3:**
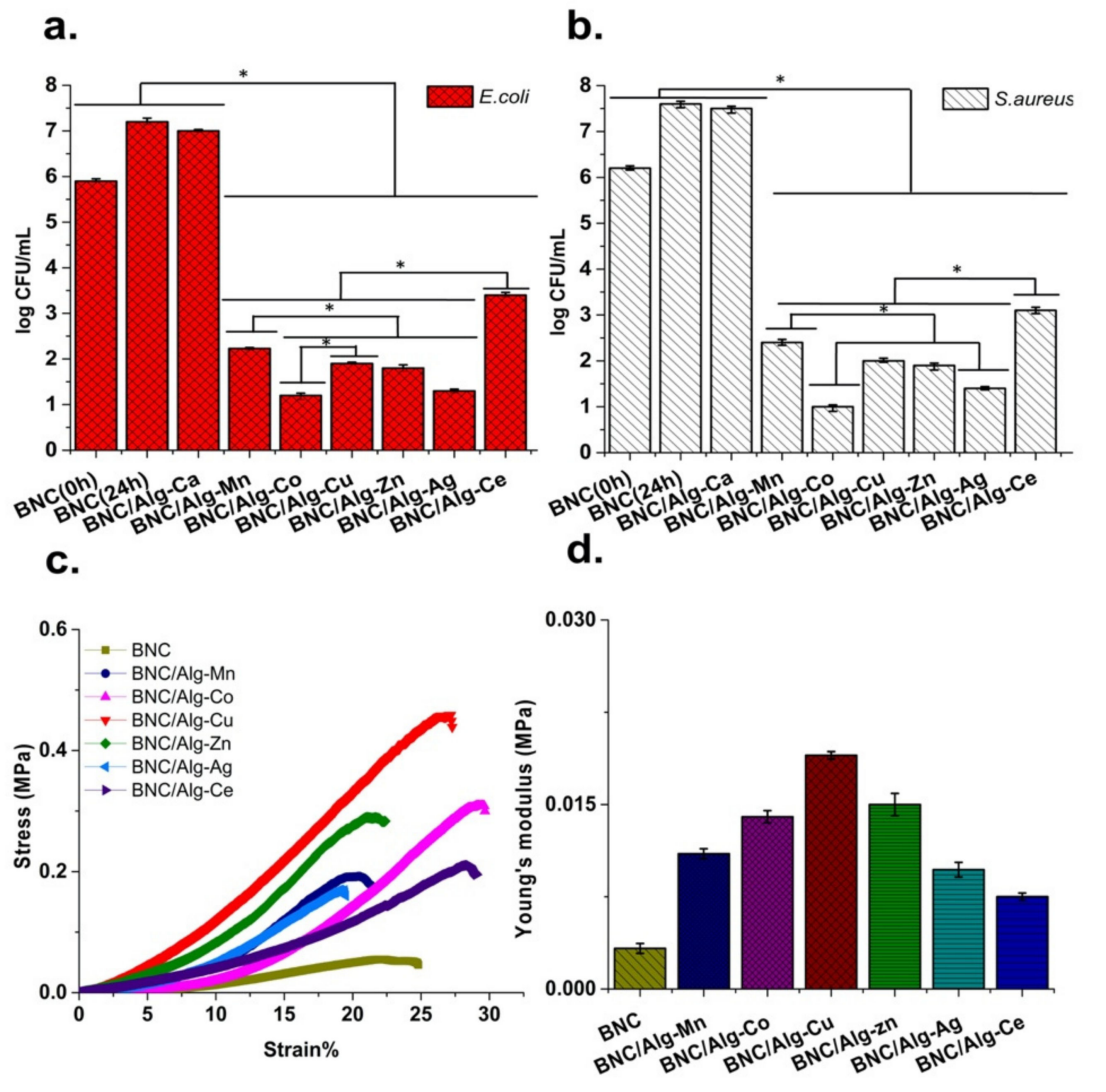
Antibacterial activity of control and synthesized dressing materials after 24 h exposure/incubation with *E. coli* (**a**) and *S. aureus* (**b**). The tensile profile (**c**) and Young’s modulus (**d**) of synthesized dressing materials in comparison to pristine BNC. * *p* < 0.05 using Student’s *t*-test (two-sided).

The results of antibacterial activity are shown in Figure 3a,b. All the synthesized dressing materials demonstrated a broader spectrum of antibacterial effect in contrast with the pristine BNC and BNC/Alg-Ca after 24 h exposure to bacterial cells. The most significant reduction in the number of viable bacterial cells was achieved with BNC/Alg-Co among all the synthesized dressings, followed by BNC/Alg-Ag > BNC/Alg-Zn > BNC/Alg-Cu > BNC/Alg-Mn > BNC/Alg-Ce. BNC/Alg-Co and BNC/Alg-Ag showed remarkable inhibition on bacterial growth for both Gram-positive and Gram-negative bacteria. This result is in accordance with the literature reporting that the antimicrobial activity of cobalt is higher than copper, followed by zinc [4]. It has been reported cerium ion is unable to penetrate mammalian cell membranes, but it has a noticeable antibacterial activity even at lower concentrations [8,43]. Wang et al. [44] reported that aqueous solutions of Cu^2+^ and Zn^2+^ at the concentration of 10^−4^ M and 10^−6^ M for Ag^+^ had antibacterial activities. Anh et al. [4] reported the minimum inhibitory concentration of aqueous solutions of Cu^2+^, Co^2+^ and Zn^2+^ was 0.004, 0.0005, and 0.002 M respectively against *E. coli*, and 0.008, 0.001, and 0.016 respectively against *S. aureus.* Kaygusuz et al. [8] reported the antibacterial activity of Alg-Ce occurred at the concentration of 0.076 M. On the other hand, Chen et al. [40] reported cross-linking of thiolated polyethylene glycol with 0.1 M AgNO_3_ is not toxic for human cells. Another research also found BNC/Alg-Chitosan composites incorporating CuSO_4_ with different concentrations (0.3, 0.1, and 0.05 M) of Cu^2+^ the viability was up to 70% [41]. After comprehensive consideration, 0.05 M was therefore chosen to ensure both hydrogel formation and the strong antibacterial activity of Algs in the study.

### 3.6. Mechanical Properties

The tensile strength and Young’s modulus of the synthesized dressing materials in comparison with the pristine BNC from 10-day static cultivation are shown in Figure 3c,d. The ultimate tensile stress of the high water-saturated pristine BNC or BNC/Alg-Na was only 0.05 ± 0.06 MPa, which was then significantly ameliorated by the introduction of cations to cross-link Alg in BNC. The mechanical property reached 0.19 ± 0.04, 0.32 ± 0.06, 0.43 ± 0.02, 0.29 ± 0.06, 0.17 ± 0.05, and 0.21 ± 0.07, respectively for BNC/Alg-Mn, BNC/Alg-Co, BNC/Alg-Cu, BNC/Alg-Zn, BNC/Alg-Ag, and BNC/Alg-Ce. The Young’s modulus of pristine BNC was 0.0033 ± 0.0004, and was enhanced in all cation cross-linked Alg/BNC composites (Figure 3d). An ideal wound dressing requires many features to protect a wound and to reconstruct the physical barrier in a wound. One of the essential features is appropriate mechanical properties, which is important for protecting a wound from mechanical damage [44]. It has been reported the tensile strength of the commercially available dressings such as Acticoat^®^, Askina^®^, and BluRibbon^®^ in wet state is in the range of 0.10–0.39 MPa [41]. The pristine BNC showed lower tensile strength than the commercial dressing materials, nevertheless after cross-linking cations, one of the obtained dressing (BNC/Alg-Cu) showed even higher tensile strength than the commercial dressings, but other synthesized dressings had similar tensile strengths as the commercial dressings.

### 3.7. pH-Responsive Release of Cations

**Figure 4:**
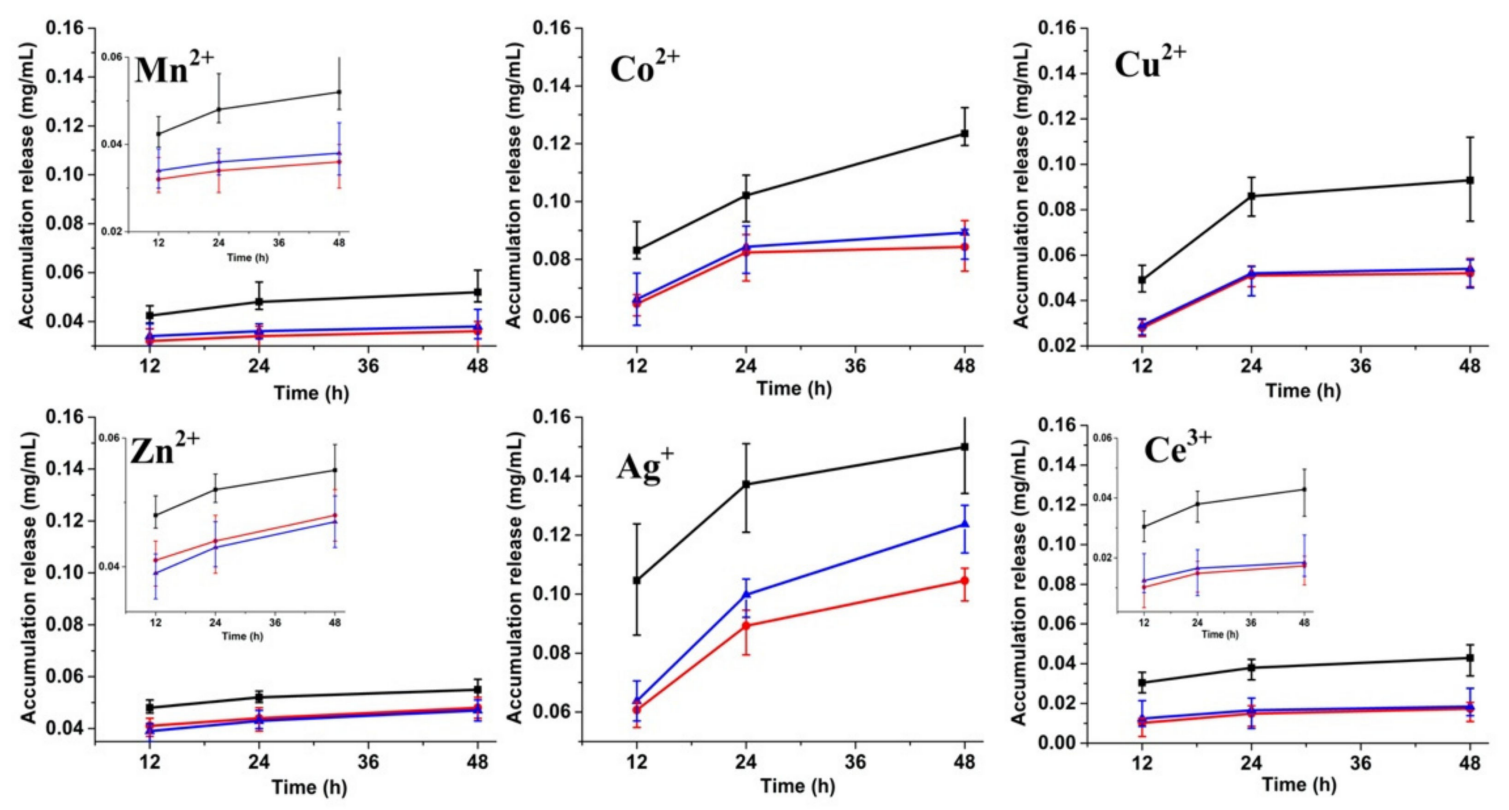
Accumulative release of metal ions from dressing materials at different pH (pH 5.5: black squares; pH 7.4: red circles, and pH 8.4: blue triangles).

As shown in Figure 4, the different cation cross-linked Alg/BNC composites showed a pH-responsive effect, which led to various release of cations. The cations were released much faster from the dressing materials at pH 5.5 in comparison to pH 7.4 and 8.4. Release of cations from BNC/Alg-Ag was faster than from BNC/Alg-Co, BNC/Alg-Cu, BNC/Alg-Zn, BNC/Alg-Mn, and BNC/Alg-Ce. It has been widely reported that the pH of a wound environment falls to the value of 5.5 due to bacterial infection, causing low-oxygen fermentation and production of lactic acid and acetic acid [4,45,46,47]. All the synthesized wound dressings indicated the capability of acting as a smart dressing in the infected wound, bringing more release of cations and further enhanced antibacterial activity. Anh et al. [4] reported that the cumulative release of cobalt was higher than zinc, which accorded with our findings. They have also confirmed the pH-responsive release of copper in which more release was observed at an acidic pH rather than natural and basic pH. The pH-dependent release of cations could be explained either by Alg hydrogel behavior at different pH or by the ions tendency to release at lower pH. Chuang et al. [18] reported that the calcium cross-linked Alg was able to release the calcium ion at lower pH (pH = 4) than at higher pH (pH = 7 and higher). Additionally, cross-linking density was reflected based on the accumulation release of cations after 48 h, which was 0.0051, 0.012, 0.0073, 0.0067, 0.014, and 0.0024 mg/cm^2^ for BNC/Alg-Mn, BNC/Alg-Co, BNC/Alg-Cu, BNC/Alg-Zn, BNC/Alg-Ag, and BNC/Alg-Ce, respectively.

### 3.8. Kinetic Analysis of Cations Release

The release profiles of cations from cross-linked Alg/BNC composites were studied using the kinetic models [29,30,31,32]. As shown in Table 2, the fitness was evaluated using the r^2^ values. In general, the release kinetics of copper fitted well with the zero-order model (r^2^ > 0.99) followed by silver and cerium (r^2^ > 0.98), cobalt and manganese (r^2^ > 0.97). Cobalt showed fitness toward both zero and first model (r^2^ > 0.97). But zinc release fitted well by first–order (r^2^ > 0.99) although it also fitted with zero-order (r^2^ > 0.98). Alg is widely used as a hydrogel matrix that shows controlled release of drugs. Bhasarkar et al. [48] have developed an Alg scaffold to deliver vitamin B12, and showed that release of vitamin B12 was fitted with first-order kinetic model (r^2^ = 0.96) and Higuchi kinetic model (r^2^ = 0.9). Additionally, Alg has an ability to respond to pH, which means higher release at lower pH. It is assumed that higher level of release can be the result of Alg deformation or shrinkage at lower pH [18]. Abdelrahman et al. [49] have reported controlled release of anti-cancer drug from Alg in an acidic environment, which was fitted with first-order (r^2^ = 0.98). Their finding is in accordance with this study where all the cations were released fitting first-order kinetic model (r^2^ > 0.97) at acidic pH.

### 3.9. Whole Blood Clotting Evaluation

**Figure 5:**
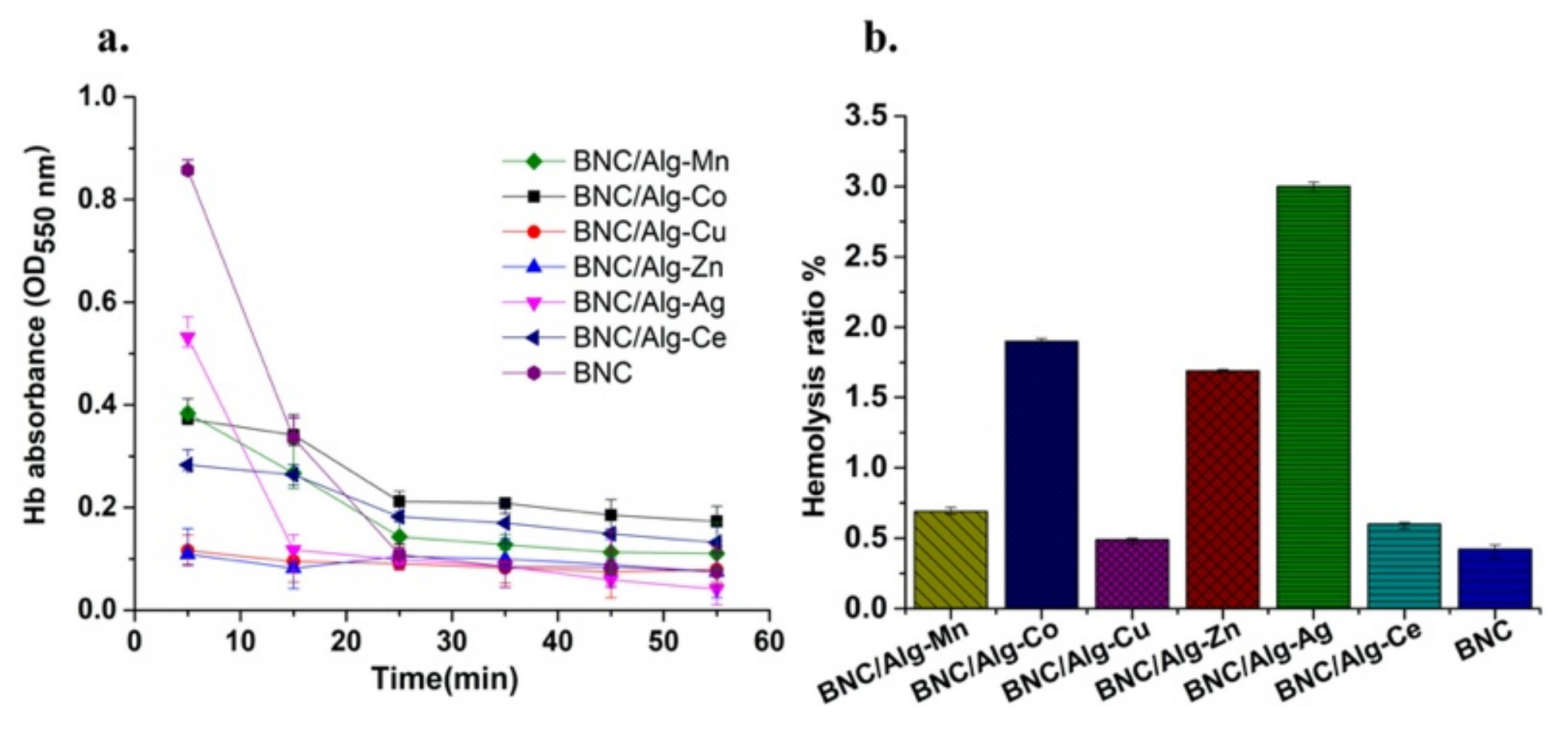
(**a**) Whole blood clotting presented by Hb (Hemoglobin) absorbance at 550 nm, (**b**) in vitro hemolytic rate of synthesized dressing materials.

The influence of synthesized dressing materials on blood clotting was evaluated by incubation with whole blood for different time duration. There revealed a reversed proportion between the absorbance value of the hemoglobin solution and the clotting rate. In other words, the high absorbance value of hemoglobin solution was associated with a slow clotting rate. As shown in Figure 5a, in the first 10 min, BNC/Alg-Zn and BNC/Alg-Cu showed the fastest clotting rate, followed by BNC/Alg-Ce, BNC/Alg-Ag, BNC/Alg-Mn, and BNC/Alg-Co. The BNC/Alg-Ag in the first 5 min had an absorbance of about 0.53, which was higher than all other materials but lower than pristine BNC. Within the first 15 min, the absorbance with BNC was higher than all composites. After 25 min contact, BNC/Al-Co, BNC/Al-Ce, and BNC/Al-Mn showed higher absorbance than pristine BNC, but the absorbance with other materials was near or less in comparison to pristine BNC (Figure 5a). The result is in accordance with the result of Tubek et al. [42], where zinc had an important role in the blood clotting process and zinc deficiency caused poor platelet aggregation and increased bleeding time in adult males.

### 3.10. In Vitro Hemolytic Rate

As shown in Figure 5b, although all the dressing materials showed absorbance below 3.1, which was favorable as a wound dressing material, BNC/Alg-Cu showed the lowest hemolysis ratio of 0.48, which was near to that of pristine BNC and following in order of: BNC/Alg-Ce < BNC/Alg-Mn < BNC/Alg-Zn < BNC/Alg-Co < BNC/Alg-Ag. These hemolysis ratio results demonstrated the excellent hemocompatibility of materials as a hemostatic agent for functional wound dressing. The hemolysis ratio indicates the leakage of hemoglobin (Hb) into the blood plasma, which is the result of the rupture extent of red blood cells (RBC) of the blood in contact with the dressing materials. The greater the hemolytic rate as a result of OD of samples are, the more broken RBCs are. Therefore, a smaller hemolytic rate as a result of smaller OD value represents the increased blood compatibility of the dressing material. It is known that the hemolysis ratio of acceptable biomaterials, required for medical applications, must be below 5 [34].

### 3.11. Cytotoxicity Test

**Figure 6:**
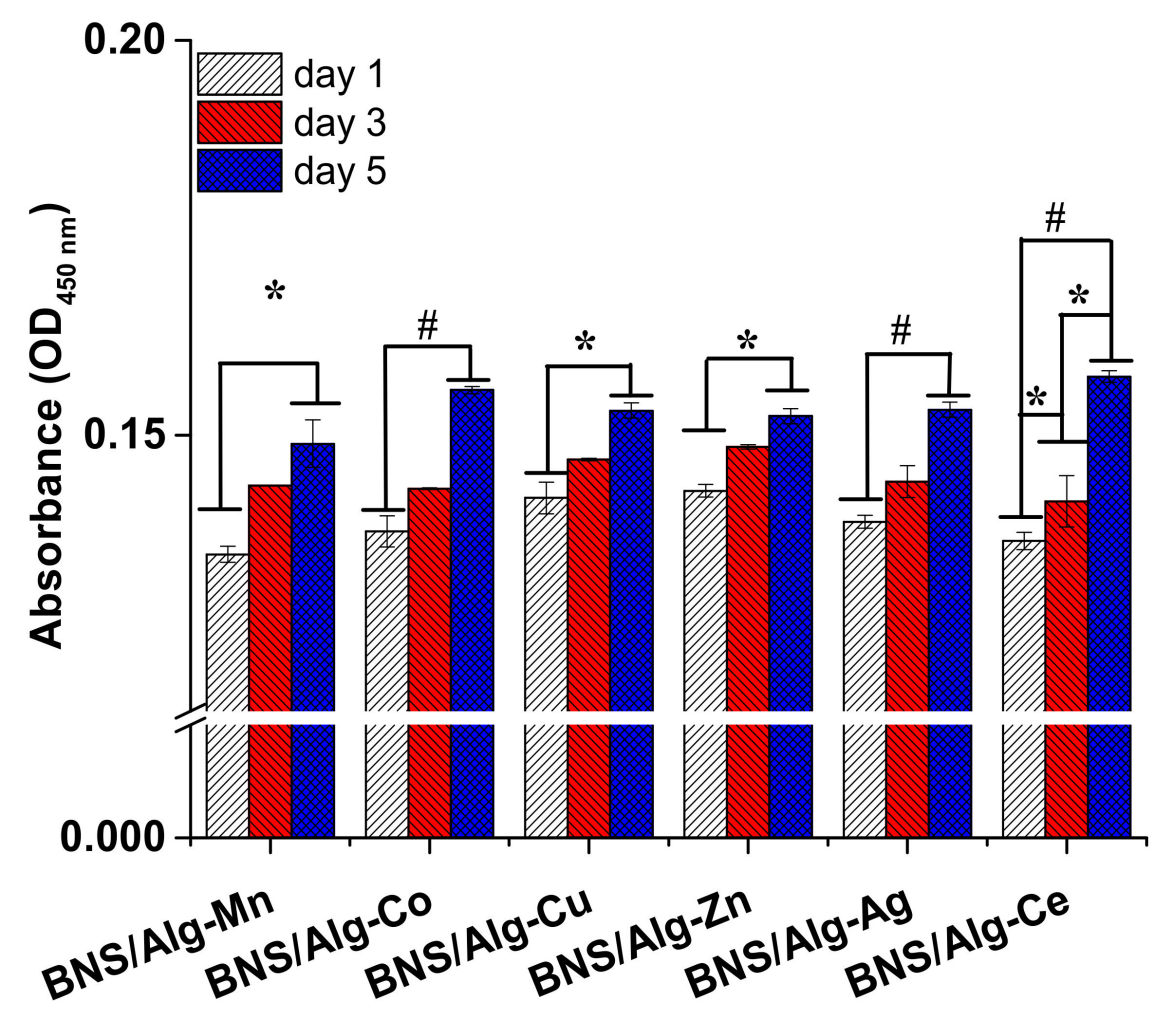
Cytotoxicity evaluation of the synthesized dressing materials using CCK-8 for L929 cells. * *p* < 0.05, # *p* < 0.001 (n = 3) using Student’s *t*-test (two-sided).

The cytotoxicity of synthesized dressing materials was investigated with L929 fibroblast cells. As shown in Figure 6, none of the dressing materials was toxic toward L929 fibroblast cells since the cells continually propagated. The growth of fibroblast cells was not negatively affected by the synthesized dressings, due to non-cytotoxicity of dressing materials and slow release of cations. After 5-d cultivation on the surface of all the six cation cross-linked dressing materials, the growth and proliferation of fibroblast cells were clearly observed in comparison to Day 1 and Day 3 cultures (*p* < 0.05). However, among the six metal cations no significant difference (*p* > 0.05) in cytotoxicity can be found within each cultivation time. Cell viability and proliferation of L929 fibroblast cells on the surface of dressing materials indicated the safety of the materials for use as functional wound dressings. Schmidlin et al. [50] reported that cerium ion had a stimulating effect on fibroblasts viability and proliferation.

### 3.12. In Vivo Healing Test

**Figure 7:**
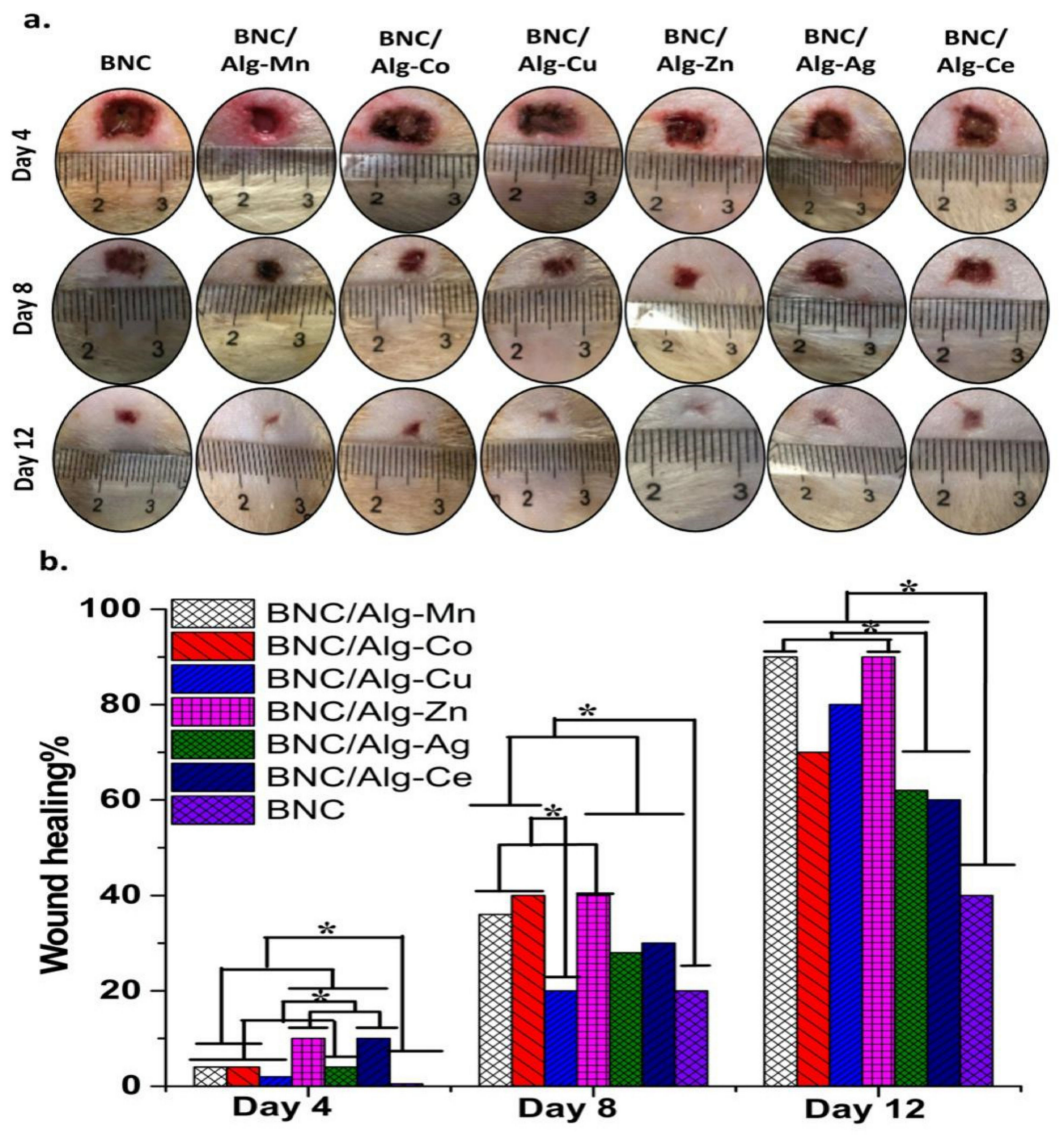
In vivo wound healing performance of the synthesized dressing materials. (**a**) Photographs of wound size during healing at 4th, 8th, and 12th day for synthesized dressing materials. (**b**) The wound healing ability of different cation cross-linked Alg/BNC composites. * *p* < 0.05 using Student’s *t*-test (two-sided).

According to the results of the in vitro study, an in vivo study was performed in a rat model. The healing ability of different materials was monitored for 12 days to heal full-thickness wound completely. The sterile synthesized dressings were applied as wound dressings to the created wounds in the dorsal area of rats, and pristine BNC was employed as a control dressing for comparison. The photos were taken periodically to evaluate healing performance over time. Reduction in wound area indicated the healing progress. As shown in Figure 7 the healing rate after eight days was more obvious for BNC/Alg-Zn. The pristine BNC showed the slowest healing rate among the tested dressing materials within twelve days. All the dressing materials could enhance the wound healing faster than pristine BNC. But the wounds covered by BNC/Alg-Mn, BNC/Alg-Zn, and BNC/Alg-Cu displayed faster recovery than other dressings, which could be suitable for application of wound dressing among all synthesized composites. Raman et al. [51] reported that zinc deficiency led to delayed wound healing due to its role in the processes of the innate and adaptive immune response, which was in agreement with this study. Zhou et al. [39] reported that Zn^2+^ cross-linked Alg could heal mouse wound faster than Cu^2+^ cross-linked Alg.

## 4. Conclusions

As expected, the six metal cations did differently affect hydrogel formation and bring in various cross-linking strength and properties, especially for antibacterial activity, hemostatic activity, and wound healing rate. The hydrogels fabricated by different cation cross-linked Algs were found to be dependent on cationic concentration, namely, the toughness of Alg hydrogels ascended with increasing cationic concentration. The physicochemical properties and biocompatibility of composites made by various valence states of cations (i.e., monovalent: silver; divalent: manganese, cobalt, copper, and zinc; trivalent: cerium) were different in vivo and in vitro. The cation concentration of 0.05 M was found to be suitable to fabricate wound dressing materials with great antibacterial activity, but further optimization to a lower concentration should be carried out. All synthesized dressing materials demonstrated improvement in WAC, WHC, and WBF, but in the case of BNC/Alg-Cu the values were close to pristine BNC, which should be explained by the higher cross-linking degree since Alg-Cu showed the toughest hydrogel (Figure 1) and the smallest level of size shrinkage in comparison to other hydrogels. The mechanical property was significantly enhanced as expected, which is essential to eliminate the requirement of a traditional secondary layer for Alg wound dressing, and to provide a promising and easy-to-handle strategy during dressing changes. The synthesized dressing materials showed the following descending sequence of antibacterial activity: BNC/Alg-Co > BNC/Alg-Ag > BNC/Alg-Zn > BNC/Alg-Cu > BNC/Alg-Mn > BNC/Alg-Ce, and they could act as smart wound dressings in an infected wound area due to the pH-responsive antibacterial activity. The release of cations was matched up to 90%, with three kinetic models commonly applied in drug release studies. All of the composite dressing materials showed better blood clotting than pristine BNC in the first 15 min of direct contact with blood, and BNC/Alg-Zn and BNC/Alg-Cu showed the fastest clotting rate. None of the synthesized composites were toxic toward fibroblast cells. In vivo skin wound healing study in an adult rat model showed significant healing when the wounds covered by the composites rather than pristine BNC, but the wounds covered by BNC/Alg-Mn, BNC/Alg-Zn, and BNC/Alg-Cu displayed faster recovery than other dressings, which could be suitable for application of wound dressing among all synthesized composites. We expect that further development will establish a smart system with cation cross-linked Alg/BNC for practical wound dressing and biomedical application.

## Figures and Tables

**Table 1:** Water holding capacity and adsorption behavior of cation cross-linked Alg/BNC composites.

Dressing Material *	WHC * (g/g)	WAC * (g/g)	WBF * (g/g)	WDWM * (g/g)
Control (BNC)	20.54 ± 0.15	15.24 ± 0.04	13.24 ± 0.03	5.14 ± 0.04
BNC/Alg-Mn	42.25 ± 0.05 ^c^	31.38 ± 0.1 ^c^	26.61 ± 0.04 ^c^	5.31 ± 0.01 ^a^
BNC/Alg-Co	23.34 ± 0.02 ^c^	19.26 ± 0.03 ^c^	17.22 ± 0.08 ^c^	8.32 ± 0.04 ^a^
BNC/Alg-Cu	20.16 ± 0.09 ^a^	17.51 ± 0.04 ^b^	14.63 ± 0.05 ^c^	6.24 ± 0.03 ^a^
BNC/Alg-Zn	23.66 ± 0.04 ^c^	21.26 ± 0.05 ^b^	20.52 ± 0.08 ^c^	15.45 ± 0.07 ^c^
BNC/Alg-Ag	49.55 ± 0.06 ^c^	46.52 ± 0.14 ^c^	37.13 ± 0.1 ^c^	5.13 ± 0,02 ^a^
BNC/Alg-Ce	37.38 ± 0.07 ^c^	33.89 ± 0.04 ^c^	31.94 ± 0.08 ^b^	15.89 ± 0.065 ^b^

* Each test was repeated eight times. Mean values ± standard deviations were given. ^a^ *p* > 0.05, ^b^ *p* < 0.05 and ^c^ *p* < 0.01, using Student’s *t*-test (two-sided). The students T-test was performed on the materials (including BNC/Alg-Mn, BNC/Alg-Co, BNC/Alg-Cu, BNC/Alg-Zn, BNC/Alg-Ag, BNC/Alg-Ce) in comparison to control (BNC).

**Table 2:** Correlation coefficients, r2, of fitting cation release according to kinetic models.

Cations	pH	Zero-Order	First-Order	Higuchi
		r^2^	r^2^	r^2^
Mn^2+^	5.5	0.9958	0.9993	0.9482
	7.4	0.9739	0.9686	0.9972
	8.4	0.9818	0.9938	9.9945
Co^2+^	5.5	0.9781	0.9714	0.9502
	7.4	0.9922	0.9994	0.9717
	8.4	0.9798	0.9819	0.9692
Cu^2+^	5.5	0.9995	0.9819	0.9963
	7.4	0.9958	0.9878	0.9839
	8.4	0.9948	0.9955	0.9981
Zn^2+^	5.5	0.9935	0.9986	0.9403
	7.4	0.9819	0.9958	0.9324
	8.4	0.9925	0.9981	0.9381
Ag^+^	5.5	0.9819	0.9958	0.9742
	7.4	0.9952	0.9162	0.9901
	8.4	0.9995	0.9695	0.9678
Ce^3+^	5.5	0.9922	0.9726	0.9624
	7.4	0.9849	0.9894	0.9869
	8.4	0.9922	0.9977	0.9945
